# Transcatheter Closure of Perimembranous Ventricular Septal Defect Using the Lifetech Konar-Multi Functional Occluder: Early to Midterm Results of the Indonesian Multicenter Study

**DOI:** 10.5334/gh.1106

**Published:** 2022-02-24

**Authors:** Rahmat Budi Kuswiyanto, Eka Gunawijaya, Mulyadi M. Djer, Noormanto, Mahrus A. Rahman, Indah K. Murni, Rubiana Sukardi, Alit Utamayasa, Rizky Ardiansyah, Ria Nova, Sri Liliyanti, Sri E. Rahayuningsih, Shirley L. Anggriawan, Tri Yanti Rahayuningsih, Dyahris Koentartiwi, Renny Soewarniaty, Venny Kartika Yantie, Sasmito Nugroho, Taufiq Hidayat, Teddy Ontoseno, Tina C. Tobing, Muhamad Ali, Muhammad Hasan Bashari, Denny S. Yosy, Nadya Arafuri, Dany Hilmanto, Piprim B. Yanuarso, Najib Advani, Sudigdo Sastroasmoro, Sukman Tulus Putra

**Affiliations:** 1 Department of Pediatrics, Hasan Sadikin Hospital/Universitas Padjadjaran, Bandung, ID; 2 Department of Pediatrics, Sanglah Hospital/ Universitas Udayana, Bali, ID; 3 Department of Pediatrics, Cipto Mangunkusumo Hospital/Universitas Indonesia, Jakarta, ID; 4 Department of Pediatrics, Sardjito Hospital/Universitas Gadjah Mada, Yogyakarta, ID; 5 Department of Pediatrics, Soetomo Hospital/ Universitas Airlangga, Surabaya, ID; 6 Integrated Cardiac Center, Cipto Mangunkusumo Hospital/Universitas Indonesia, Jakarta, ID; 7 Department of Pediatrics, Adam Malik Hospital, USU, Medan, ID; 8 Department of Pediatrics, M. Hoesin Hospital/Universitas Sriwijaya, Palembang, ID; 9 Department of Pediatrics, Moewardi Hospital/UNS, Surakarta, ID; 10 Department of Pediatrics, Eka Hospital, Pekanbaru, ID; 11 Department of Pediatrics, Chasbullah Abdul Madjid Hospital, Bekasi, ID; 12 Department of Pediatrics, Saiful Anwar Hospital/ Universitas Brawijaya, Malang, ID; 13 Department of Pharmacology and Therapy Faculty of Medicine Universitas Padjadjaran, Bandung, ID

**Keywords:** Perimembranous ventricular septal defect, transcatheter closure, KONAR-multi functional occluder

## Abstract

**Background:**

The alternative device to close perimembranous ventricular septal defect (pmVSD) has been searched for better result, less complications and applicable for infants. However, the ideal device is still unavailable. We aimed to evaluate the effectiveness and outcome of transcatheter pmVSD closure using the KONAR-multi functional occluder (MFO).

**Methods:**

Clinical, procedural, follow-up data of pmVSD patients with symptom of heart failure or evidence of significant left to right shunt, growth failure, recurrent respiratory tract infection, and history of endocarditis who underwent transcatheter closure using the MFO were prospectively evaluated.

**Results:**

Between January 2016 and December 2017, there were complete records of 132 pmVSD children closed using MFO from eleven centers in Indonesia. The median of age was 4.5 (0.3–17.4) years; weight 14.8 (3.5–57) kg, defect size at the smallest part 3.4 (1.0–8.1) mm, flow ratio 1.6 (1.3–4.9), mean pulmonary artery pressure 18 (7–79) mmHg, fluoroscopy time 18 (3.8–91) and procedural time 75 (26–290) minutes. A retrograde approach was done in 41 (31%) patients. Procedures succeeded in first attempt in 126 (95.4%), failed in three and migration in three patients. Six of eight infants with congestive heart failure were closed successfully. Of 126 patients with successful VSD closure, 12 months follow-up were completed in all patients. The rate of complete occlusion at 1 month, 3 months, 6 months and 12 months after intervention were 95.2%, 97.6%, 99.2%, and 99.2%, respectively. New-onset aortic regurgitation and moderate tricuspid regurgitation developed only in five and three patients. Neither complete atrioventricular block, nor other complications occurred.

**Conclusion:**

Transcatheter closure of pmVSD using the MFO is safe, effective, and feasible in infants and children.

## Introduction

An early experience of transcatheter perimembranous ventricular septal defect (pmVSD) closure with Amplatzer membranous occluder initially gave enthusiasm [1,2]. However, subsequent studies reported higher rates of early to late post-procedural atrioventricular heart block (AVB) compared to surgery [3,4,5,6,7]. The widespread use of this device was abandoned in many centers afterwards. Despite this controversy and not being approved in many countries, transcatheter pmVSD closure continues to be performed, including in Indonesia using various modified devices or other devices originally designed for other lesions, such as VSD device occluder from China [8,9,10,11], Nit-Occlud Lê-VSD-Coil (Cologne, Germany) [12,13], Amplatzer Duct Occluder (St. Jude Medical Inc., United States), Cocoon Duct Occluder (Vascular Innovations Co. Ltd., Thailand), and Cera Duct Occluder (Lifetech Scientific Co. Ltd., China) [14,15], and Amplatzer Duct Occluder II (ADO II) (St. Jude Medical Inc., United States) [16,17]. However, the efficacy has varied and possible complications such as complete AVB, hemolysis, new onset aortic regurgitation (AR) and tricuspid regurgitation (TR) continues occurring, although complete AVB occurrences were less frequent compared to the first generation of the Amplatzer membranous occluder. Furthermore, the applicability of transcatheter closure of pmVSD in infants with heart failure is limited due to the high proportion of failure and complications [15,18]. The perfect devices to close pmVSD have been searched for better results aiming to lower the complication rates. Meanwhile, an ideal device to close pmVSD is still unavailable. Accordingly, this study aimed to evaluate the effectiveness and safety of the KONAR-Multi Functional Occluder (MFO; Lifetech Scientific, Shenzhen, China) as a novel device to close pmVSD.

## Material and Methods

### Patient Selection

A prospective study was conducted to evaluate transcatheter closure of pmVSD using the MFO in eleven centers in Indonesia from January 2016 to December 2017 under collaboration of the Cardiology Working Group of the Indonesian Pediatric Society. Indications for closure were defects with heart failure symptoms or evidence of significant left to right shunt, including left heart enlargement on transthoracic echocardiography (TTE), cardiothoracic ratio >0.5, or pulmonary to systemic blood flow ratio >1.5, growth failure, recurrent respiratory tract infections [19], and a history of endocarditis. Patients with pulmonary hypertension with right to left shunt or pulmonary vascular resistance >8 wood units, iliac vein thrombosis, active endocarditis, or other cardiac defects requiring surgery were excluded. Informed consent was obtained from all parents or patients prior to the procedure. The study was approved by the Ethics Committee of the hospitals.

### Device Description

The MFO is a self-expandable, double-disc device made from a layer nitinol wire mesh with 144 wires of 0.002” nitinol cables. Both discs are linked together by a cone-shaped waist. The left disc attached to the base of this cone. An umbilicated arm joins the vertex of the left disc and the right disc and allows articulation to the right disc. The left disc or ‘high-pressure disc’ is attached to the base of the truncated cone of the waist, and the right disc (D1) or ‘low-pressure disc’ is attached to the waist arm. Each disc contains a 2.4 mm long hub with a screw so that the device can be positioned retrogradely or anterogradely. Both discs of the device are of equal size (a symmetric device). The base of the cone or ‘D2’ has a diameter of 2 mm greater than the vertex. adding 2 mm on each side to D2.’ The diameter of the left disc labelled by ‘D.’ The right disc is more flexible for lower pressure with the same range in diameter size of the left disc. The waist of the last four large models (diameters ‘D’ is ≥14 mm) are sewn with polytetrafluoroethylene (PTFE) membranes securely using nylon threads in order to increase its occlusion ability and reduce the residual shunts, while the other four small models have no membrane in it. One screw hub is made on each disc. (***Image/Figure 1*** and ***Table 1***).

**Image/Figure 1:**
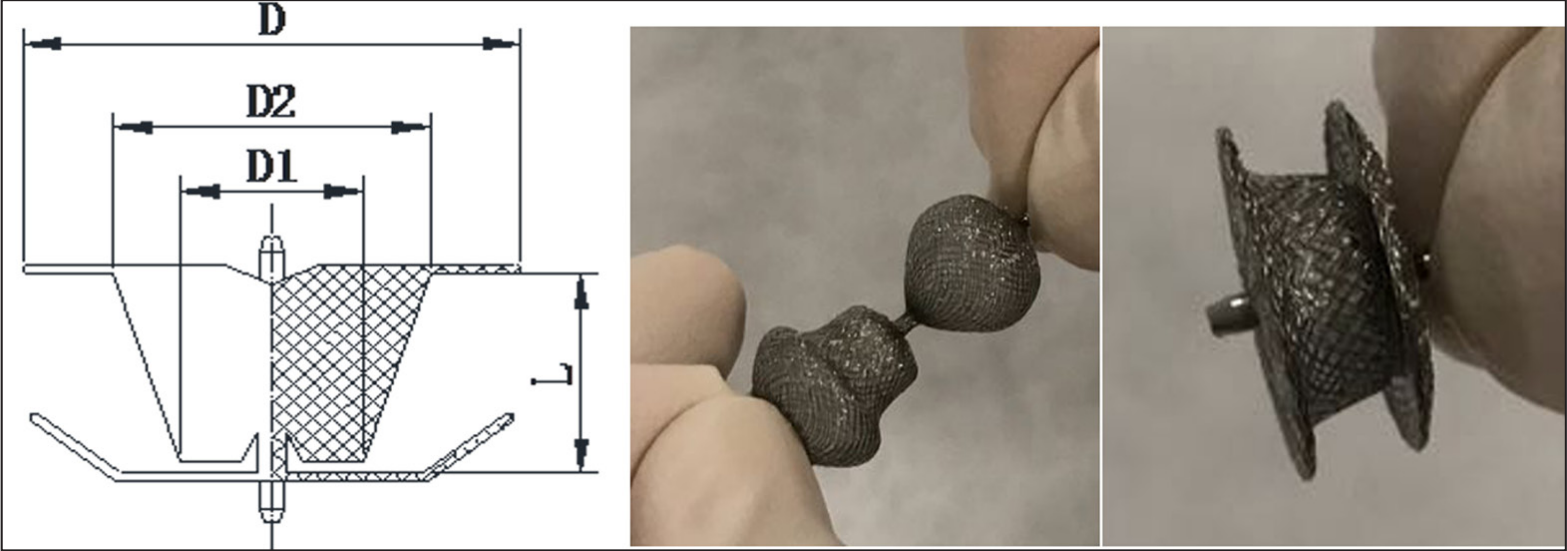
The KONAR-Multi Functional Occluder. Device components denomination: D. Disc diameter; D1. Waist Diameter Right Ventricular side; D2. Waist Diameter Left Ventricular side.

**Table 1:** The Konar-multi functional occluder specification.


SIZE	D*	D1*	D2*	L*	DELIVERY SHEATH (F)	PATIENTS N, %

5–3	10	3	5	4	4–5	18 (13.6)

6–4	10	4	6	4	4–5	38 (28.8)

7–5	12	5	7	4	5	26 (19.7)

8–6	12	6	8	4	5	25 (18.9)

9–7	14	7	9	4	6	12 (9.1)

10–8	14	8	10	4	6	8 (6.2)

12–10	16	10	12	4	7	4 (3.0)

14–12	18	12	14	4	7	1 (0.7)


D = Diameter of retention disc; D1 = right waist diameter, D2 = left waist diameter, L = total length of the device, *in millimeter, N = 132.

### Procedural Closure

The procedure was performed under general or local anesthesia, with TTE guidance. Access was obtained via the femoral vein and femoral artery. Heparin (75–100 IU/kg) and cephazolin or ampicillin (50 mg/kg/iv) was administered 30 minutes before procedure. The hemodynamic study was performed prior to closure. Left-ventriculography on the long axial oblique or 4-chambers view was performed using pigtail catheter to assess the defect. The size of VSD was measured at the left ventricle (LV) and right ventricle (RV) orifices. The device size (D1) was chosen 1–2 mm larger than the smallest part of the defect. The defect was crossed from the LV or occasionally from the RV with a 0.032” or 0.035” J-angled tip Terumo exchanged guide wire via a 4/5F Judgkin Right (JR) or cut pigtail catheter to establish a delivery catheter access.

Antegrade approach modified from the previous study [1,2,3], was performed by establishing the arterio-venous loop. The Terumo exchange wire was snared in the pulmonary artery or superior vena cava. Subsequently, delivery sheath was advanced with kissing technique from the femoral vein through the defect until it reached the LV cavity or descending aorta. The left disc was deployed in the LV cavity or ascending aorta and pulled back and positioned into the defect, followed by deploying the waist and right-disc.

The retrograde approach modified from a previous study [16], was performed without establishing arterio-venous loop and considered if the distance between the defects and aortic valve was ≥3 mm. A 2.0 mm must be added on each side of the LV waist (D2) to obtain the diameter of the left disc (D), except for the 5–3, 7–5 and 9–7 devices a 2.5 mm should then be added on the D2. Therefore, it is safe enough to implant the device with this requirement to avoid the left disc (D) compressing the aortic valve with a minimum aortic rim is 3 mm.

In case of the left disc (D) interfered the aortic valve, we selected the size of the device that was similar to the narrowest part of the VSD size, not to exceed this measure by 1 mm. When there was an aneurysm, we selected the device size as similar to the narrowest part of the VSD, but not to exceed this diameter by 2 mm. In case of multiple exits, we selected the device that should cover the left ventricle side of the defect.

An antegrade approach was considered in small patients with relatively large defect and who need larger sheath, or in case of deficient aortic rims or the distance between the defect to aortic valve was insufficient, or there was trivial aortic regurgitation or mild aortic valve prolapse.

The delivery sheath was advanced over the wire directly from the aorta until reached RV cavity. RV disc was deployed and positioned on the defect, followed by deploying the LV disc.

Left-ventriculography and TTE were repeated before and after releasing the device to assess the device position, closure result and competency of the valves (***Image/Figure 2***). When there was complete closure, or only smoky or non-significant residual shunt through the device, or no new onset of aortic and tricuspid regurgitation or obstruction, we then proceeded to release the device. After closure, aspirin 3–5 mg/kg daily for six months was started and endocarditis prophylaxis was advised if needed until complete closure was documented.

**Image/Figure 2:**
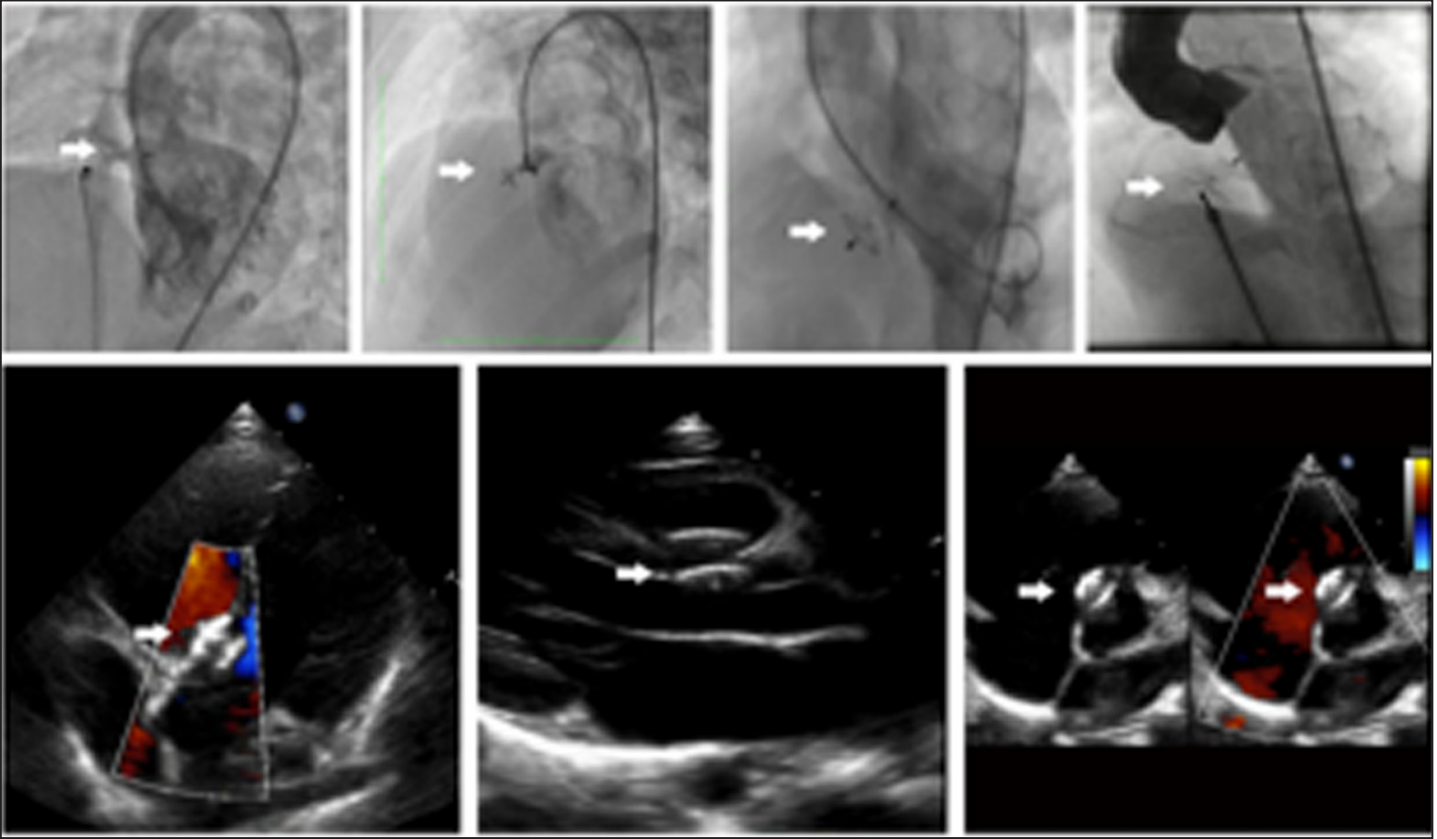
Angiography and echocardiography after closure.

We did complete clinical examination, 12-lead electrocardiography, and TTE at prior to discharge, 1-month, 3-months, 6-months, and 12-months after procedure.

### Outcome measures

Procedural success was defined by device release in the appropriate position without embolization. Immediate complete closure was determined by angiography shortly after deployment of the device. TTE was performed 24 hours after intervention or prior to discharge to determine the occlusion status for every patient and considered for the outcome at discharge.

The residual shunt was classified as into trivial, small, moderate and large shunts. A trivial shunt was defined when the width of the color jet as assessed by color Doppler echocardiography as it exited the septum of <1 mm. Small, moderate, and large shunts were considered when the width of 1 to 2 mm, 3 to 4 mm, or width ≥4 mm, respectively [3].

A valve regurgitation was considered when persistent new or increased valvar regurgitation occurred after the procedure. A heart block was considered when there was a complete atrioventricular block or other persistent cardiac arrhythmia that required long-term medical treatment or pacemaker placement [3].

### Data Collection

Protocol was distributed via e-mail among all participating centers to standardize the collected clinical, procedural and follow-up data, using a detailed instruction sheet. The data were collected at each center, submitted and compiled by the principal investigator, including the clinical baseline, defect profile, hemodynamic and procedural, closure result and follow-up data. When data were inconclusive or required additional data, the local investigator was directly contacted to clarify.

### Data Analysis

Continuous variables are presented as mean with standard deviation or median with interquartile range for normally distributed and skewed data, respectively. Categorical variables are presented as frequencies and percentages. Normality of the variables was checked using Kolmogorov-Smirnov test. T-test or Wilcoxon test was used to assess the difference of variables. A two-sided *p*-value < 0.05 was considered statistically significant. Data were analyzed by using SPSS Statistics for Windows, Version 23.0 (IBM SPSS Statistics for Windows, Version 23.0. Armonk, NY: IBM Corp).

## Results

### Subject Characteristics

Between January 2016 and December 2017, there were 195 pmVSD patients who underwent transcatheter closure using MFO at eleven centers in Indonesia. Data that were not received or incomplete for 55 and 8 adult patients were also excluded, so that only 132 patients were included in the study (***Table 2*** and ***Image/Figure 3***). Eight patients were infants with clinically congestive heart failure who did not respond to medical therapy (***Table 3***). Concomitant anomalies were small atrial septal defect in four patients, tiny patent ductus arteriosus, mild pulmonary valve stenosis, bicuspid aortic valve, and mild aortic valve stenosis, each found in one patient. One patient had a history of residual defect post coil VSD closure with persistent severe hemolysis. Membranous septum aneurysm was found in 46 patients (34.8%) and multiple exit defects in 19 patients. Thirteen patients with flow ratio below 1.5 were closed due to frequent respiratory tract infections in 4, history of endocarditis in 3, growth failure in 4, and psychosocial issues in 2 patients (***Table 4***).

**Table 2:** Subject characteristics and procedural data.


CHARACTERISTICS AND PROCEDURAL DATA (N = 132)	MEDIAN	MINIMUM	MAXIMUM	1^ST^ QUARTILE	3^RD^ QUARTILE	IQR

Age in years	4.5	0.3	17.4	2.5	7.2	4.6

Body weight in kilogram	14.8	3.5	57.0	10.0	20.2	10.2

Defect size on LV in mm	4.5	1.0	11.0	3.3	6.3	3.0

Defect size on RV in mm	3.4	1.0	8.1	2.4	4.4	2.0

Flow ratio	1.6	1.3	4.9	1.5	1.8	0.3

Mean pulmonary arterial pressure in mmHg	18.0	7.0	79.0	13.7	23.0	9.3

Fluoroscopy-time in minute	18.0	3.8	91.0	12.0	25.5	13.5

Procedure-time in minute	75.0	26.0	290.0	60.0	92.0	32.0


LV, left ventricle; IQR, interquartile range; RV, right ventricle.

**Image/Figure 3:**
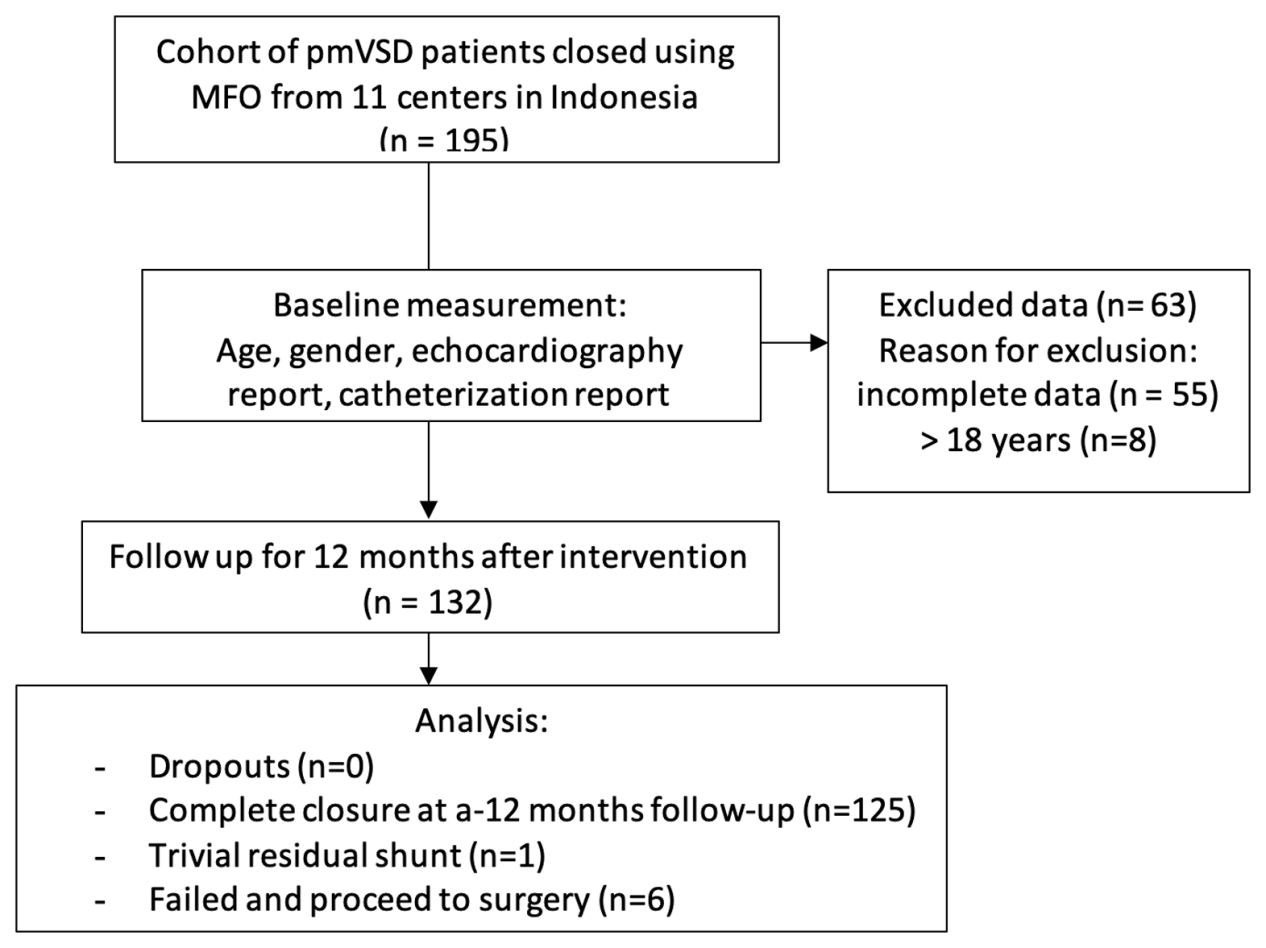
Flow chart of transcatheter in ventricular septal defect closure of a multicenter study in Indonesia.

**Table 3:** Baseline characteristic and procedural data of infants undergoing transcatheter VSD closure (n = 8).


BASELINE AND PROCEDURAL DATA	

Age (months), Min^a^, Median, Max^b^ (IQR^c^)	3.3, 6.3, 12.0 (6.3)

Male sex, n (%)	2 (25)

Body weight (kilogram), Min^a^, Median, Max^b^ (IQR^c^)	3.5, 4.8, 8.0 (1.8)

Defect size on LV (mm), Min^a^, Median, Max^b^ (IQR^c^)	5.9, 8.1, 9.8 (1.6)

Defect size on RV (mm), Min^a^, Median, Max^b^ (IQR^c^)	3.0, 5.2, 8.1 (3.8)

Septal aneurysm, n (%)	2 (25)

Flow ratio, Min^a^, Median, Max^b^ (IQR^c^)	1.6, 2.4, 4.9 (0.7)

Mean pulmonary arterial pressure (mmHg) Min^a^, Median, Max^b^ (IQR^c^)	23, 32.5, 79 (14.3)

Fluoroscopy time (minute), Min^a^, Median, Max^b^ (IQR^c^)	16.8, 28.1, 91.0 (7.6)

Procedure time (minute), Min^a^, Median, Max^b^ (IQR^c^)	70, 131.5, 290 (71.7)

Device size; n (%)	

6–4	1 (12.5)

8–6	4 (50)

12–10	2 (25)

14–12	1 (12.5)


Min^a^, Minimum. Max^b^, Maximum. IQR^c^, Inter Quartile Range.

**Table 4:** Indications for Intervention.


INDICATIONS	NUMBER OF CHILDREN (%)

Pulmonary to systemic flow ratio (Qp/Qs) ≥ 1.5	101 (76.5)

Pulmonary to systemic flow ratio more than 1.5 with growth failure	18 (13.6)

Pulmonary to systemic flow ratio (Qp/Qs) < 1.5 with additional indication:	

Growth failure	4 (0.7)

Recurrent respiratory tract infections	4 (0.7)

Psychosocial indication	2 (1.5)

History of endocarditis	3 (2.3)


Qp/Qs, pulmoary flow/systemic flow.

### Procedural Data

Out of 132 patients, 121 (91.7%) patients had successful implantation on first attempt, seven on second attempt, and more than twice in one patient. The device was implanted with retrograde approach in 41 cases (31%). Angiography after device deployment revealed that complete closure occurred in 68.2% patients. The other patients still had trivial, small, moderate and large residual shunts (22.7%, 4.5%, 2.3% and 2.3%, respectively).

Failure to close the VSD occurred in three cases (2.3%); which were related to the floppy rim underneath aortic valve in a 2-year-old girl with Down syndrome, large defect and aortic impairment in 9-year-old boy, and unavailable appropriate device size in a 3.3-month-old infant. Closure of the device was aborted directly in these three patients with large residual shunt and all of the devices were retrieved directly.

Device migration occurred in three cases: 3-year-old boy with Down syndrome, 3-year-old with pre-existing mild aortic valve prolapse and mild AR, and a 3.5-month-old infant. These cases required multiple attempts to implant and showed moderate residual shunt on the immediate result. These migrations occurred several minutes after releasing device in one case and on the next day after procedure in two cases. All devices were percutaneous retrieved successfully. We referred all of the failed cases for surgical closure at the same hospitals.

The occlusion rate at discharge was 82.6% (***Table 5***). The fluoroscopy-time and procedure-time were varied widely related to the prolonged procedure in infant, multiple attempted case, and retrieval of the device. The procedure-time and fluoroscopy-time were reduced significantly using the retrograde approach (***Table 6***).

**Table 5:** Short term outcome of transcatheter closure of perimembranous ventricular septal defect using the Lifetech Konar-multi functional occluder stratified by age of patients (N = 132).


	COMPLETE CLOSURE N (%)	RESIDUAL SHUNT N (%)				MIGRATION N (%)	FAIL TO CLOSE N (%)

		TRIVIAL	SMALL	MODERATE	LARGE		

Immediate result							

<1 year	0 (0)	4 (3.0)	2 (1.5)	1 (0.7)	1 (0.7)	0 (0)	0 (0)

1–5 year	48 (36.4)	15(11.4)	1 (0.7)	2 (1.5)	1(0.7)	0 (0)	0 (0)

5–18 year	42 (31.8)	11(8.3)	3 (2.3)	0 (0)	1 (0.7)	0 (0)	0 (0)

Total	90 (68.2)	30(22.7)	6 (4.5)	3 (2.3)	3 (2.3)	0 (0)	0 (0)

Result at discharge							

<1 year	3 (2.3)	1 (0.7)	2 (1.5)	0 (0)	0 (0)	1 (0.7)	1 (0.7)

1–5 year	58 (43.9)	6 (4.5)	0 (0)	0 (0)	0 (0)	2 (1.5)	1 (0.7)

5–18 year	48 (36.4)	8 (6.0)	0 (0)	0 (0)	0 (0)	0 (0)	1 (0.7)

Total	109 (82.6)	15(11.4)	2 (1.5)	0 (0)	0 (0)	3 (2.2)	3 (2.3)


**Table 6:** Comparison of transcatheter in ventricular septal defect closure approach.


	ANTEROGRADE	RETROGRADE	P VALUE

Number of patients, n (%)	91 (68.9)	41 (31.1)	0.001

Weight in kg, mean (SD)	16.3 (9.5)	18.3 (10.4)	0.28

≤10 kg, n (%)	26 (28.6)	8 (19.5)	0.29

>10 kg, n (%)	65 (71.4)	33 (80.5)	

Age in years, mean (SD)	5.2 (3.6)	5.5 (4.1)	0.62

Fluoroscopy time, mean (SD)	23.7 (16.8)	18.1 (12.5)	0.05

Procedure time, mean (SD)	89.5 (40.2)	69.4 (27.8)	0.004


SD, standard deviation.

### Complications

Transient first-degree AVB developed in two patients; transient supraventricular tachycardia and ventricular tachycardia developed each in one patient during procedure and resolved spontaneously without additional intervention. Right bundle branch block, hypotension and fever occurred, which were resolved prior to the hospital discharge, each in one patient. No complete AVB, hemolysis or death during the procedure and follow-up were found (***Table 7***). The majority of patients (96%) were discharged well on the following day after the procedure. Three patients required pack red cell transfusion. Packed red cell transfusion was required in these three patients because the level of baseline hemoglobin before procedure in these three patients was low and getting lower after the procedure, although no significant blood loss during procedure occurred. None of them required intensive care admission.

**Table 7:** Short term complication of transcatheter in ventricular septal defect closure stratified by age of patients (N = 132).


COMPLICATION	N = 132 (%)

Death	0 (0)

Hemolysis	0 (0)

Ventricle Fibrillation	

<1 year	1 (0.7)

First degree AV block	

<1 year	2 (1.5)

5–18 year	1 (0.7)

Right Bundle Branch Block	

1–5 year	1 (0.7)

Total AV block	0 (0)

Supraventricular tachycardia	

<1 year	1 (0.7)

Systemic hypotension	

<1 year	1 (0.7)

1–5 year	1 (0.7)

Fever 24 hour after intervention	

1–5 year	1 (0.7)

Mild Aortic Regurgitation	

1–5 year	2 (1.6)

Trivial Aortic Regurgitation	

<1 year	2 (1.6)

1–5 year	6 (4.5)

5–18 year	5 (3.7)

Moderate Tricuspid Regurgitation	

<1 year	3 (2.3)

1–5 year	1 (0.7)

Trivial to Mild Tricuspid Regurgitation	

<1 year	1 (0.7)

1–5 year	7 (5.3)

5–18 year	6 (4.5)


AV, arteriovenous.

### Follow-up results

Of 126 patients with successful VSD closure, 12 months follow-up were completed in all patients (***Figure 3***). The rate of complete occlusion at 1 month, 3 months, 6 months and 12 months after intervention were 95.2%, 97.6%, 99.2%, and 99.2%, respectively (***Table 8***).

**Table 8:** Long term outcome of transcatheter in ventricular septal defect closure using the Lifetech Konar-multi functional occluder in Indonesia.


OUTCOME	1 MONTH	3 MONTHS	6 MONTHS	12 MONTHS

Complete closure, n (%)	120 (95.2)	123 (97.6)	125 (99.2)	125 (99.2)

Trivial residual shunts, n (%)	6 (4.8)	3 (2.4)	1 (0.7)	1 (0.7)


Mild and trivial aortic regurgitation that occurred shortly after released of the device were 1.6% (2 patients) and 10.3% (13 patients), respectively. The aortic regurgitation was resolved spontaneously at 12 months follow-up with percentage of trivial regurgitation of 10.3% (13 patients) and mild regurgitation of 0.7% (1 patient). New onset of AR developed in five patients; at the end of follow-up four patients are with trivial AR and one with mild AR.

The TR occurrences were assessed by TTE to detect for oversized occluder and regurgitation pressure gradient. Moderate TR occurred in four patients (3.2%) shortly after releasing devices, in which three of them were infants and related to the oversize of the right ventricular disc. One patient with moderate TR occurred due to the malposition of the device to the tricuspid valves. However, at a 12-month follow-up, echocardiography results showed the improvement of the regurgitation. Trivial and mild TR occurred in 12 patients (9.5%) (***Table 8***). None of these patients required additional treatment. During the follow-up, these patients were clinically well and the regurgitation was decreasing despite persisting, and were in the mild degree at the latest follow-up. None of these patients required additional treatment.

## Discussion

### Procedural success

This study demonstrated a high procedural success rate (95%) of transcatheter pmVSD closure using the Konar-Multi Functional Occluder (MFO). The procedural success rate was comparable to previous studies using different devices, such as Amplatzer membranous occluder was 89–99% [3,4,5,6,7], Le VSD coil was 91–94% [12,13], ductal occluder was 90–96% [14,15], and modified device from China was 95–98% [8,9,10,11], respectively.

All failure of procedures and device migration that occurred during the early phase of this study were associated with incorrect selection of the patient and technical error in the use of this new device. Afterwards, based on the early experiment, several factors should be considered such as technical understanding of the device and the operator experience, procedure in small patients or infants, patients with large defect or pre-existing aortic insufficiency, leading to a high risk for device embolization.

The Konar-MFO is easy to retrieve if being embolized because it has a removable screw on the left and right disc and the flexibility property, which provide lower profile and smaller delivery sheath. The Konar-MFO can be introduced through varieties of the catheters or smaller profile of 4-7F delivery system, such as Mullin long sheath, JR guiding catheter or other delivery sheath. More than 90% of implantation were on the first attempts. These contributed to the unique characteristic of the user friendly and reproducible device of the Konar-MFO, and offered advantages to membranous VSD occluder, duct occluder and Le VSD coil. Additionally, our series showed most cases required single device selection, and only five patients needed a second larger device on more than one attempted procedure.

In this study, the fluoroscopy time and procedure time were comparable to other studies [4,17]. Prolonged procedure was required in migrated device and multiple attempted cases, and any procedure in infants, but it was not related to the specification of the device. The international pmVSD registry using the Amplatzer membranous reported longer fluoroscopy and procedure time in lower weight patients [4]. In this study, the device was successfully implanted with retrograde approach in 41 cases (31%). Retrograde approach could reduce radiation exposure (*p* = 0.05) and shorten procedure time (*p* = 0.004). However, this retrograde approach should not be used in small children or defect with bald aortic rim to avoid aortic valve injury. The exposure time of the retrograde approach was shorter and the therapy was cost-effective compared to the antegrade approach [17]. Compared to ADO II, the Konar-MFO can be used for the larger defect, either antegrade or retrograde without changing the position of the left or right disc.

### Residual shunt

In this study, direct effectiveness was lower with less than quarter of patients having smoky-small residual shunt at immediate result. This may relate to a delay in closure process, as the first-fourth size of MFO does not contain a PTFA membrane to enhance the clotting process. Smokey residual shunt through the device was acceptable since the residual was non-hemodynamically significant. The complete closure rate increased to 98% at the end of follow-up period. The proportion of complete closure increased from the immediate result to the latest follow-up using Amplatzer membranous occluder from 47% to 99% [3,4,5,6,7], ductal occluder from 66 to 96–99% [14,15], and ADO II from 78 to 94% [16,17]. The residual shunt using modified Chinese device occluder was reported 7% [8,9,10,11], and using Le VSD coil reported 18–34% [12] and complete closure was achieved in 99% cases in the European registry [13].

### Closure in infants

Previous studies reported transcatheter pmVSD closure in infants and small children had lower procedural success and higher major complication rates [4,5,6,18]. A lower weight of the patient was significantly correlated to increased risk of procedure or device-related complications and residual shunts [4,15]. Selection of the patients, operator experience, size of defect and size of the delivery sheath are important considerations to close in infants [4,15].

With the distinct features of the Konar-MFO, six of eight patients in this study were infants with clinically overt congestive heart failure not responding to medical therapy, were successfully and safely treated without surgery despite requiring longer procedure time. However, three infants showed moderate tricuspid regurgitation due to the length of the device relatively longer in infants, which interfered with the tricuspid valve. The implantation was decided despite moderate TR, since the device was well-positioned with a tiny to small non-significant shunt. These patients were clinically well and improved, and the degree of TR reduced on further follow-up. New onset TR may relate to relatively larger size of right-disc and the length of device, especially when stretching, or the small waist becoming entangled easier with the leaflet of the tricuspid valve or protruding right disc which might interfere with the valve movement. Pushing the cable toward RV apex can reduce tricuspid valve involvement. In our opinion, to avoid this complication, the size of the right-disc should be smaller and the length should be shorter, especially for small patients. Our results showed the feasibility of MFO and its low profile and flexible delivery system to use in selected infants with pmVSD and congestive heart failure.

### Aortic insufficiency

The proportion of new-onset AR following transcatheter closure of pmVSD was reported up to 16% using ductal occluder [14,15], and up to 17% using Amplatzer membranous occluder [4,5,6,7]. The proportion of new-onset AR was lower using ADO II [16,17], and modified Chinese occluder [8,9,10,11]. In a study using Le VSD Coil, the proportion of AR was higher, but they included the patients with pre-existing aortic insufficiency [12]. The subsequent study reported lower incidence of AR [13].

The location of pmVSD is adjacent to the tricuspid and aortic valve. The device implantation may affect the surrounding valve, causing valve insufficiency. The sufficient distance between the defect and the aortic valve is essential to avoid aortic regurgitation. The ideal distance from the defect to the aortic valve using the Konar-MFO is more than 3 mm. The softer and flexible device, such as Le VSD coil or ADO II could be used in defects with aortic rims 3 mm or less. A small number of studies reported that pmVSD without aneurysm is difficult to be treated with the Konar-MFO as the symmetrical discs might injure the aortic valve [21]. However, our series showed a good feasibility as 65.2% of our patients were without aneurysm. New-onset AR was less compared to TR, commonly related to the left disc configuration that made it less stiff and flexible, the size of the device and the distance between the VSD upper rim and the aortic valve leaflet. The development of AR was influenced not only by the type of the device and defect variation, but also by the number of the subjects and the experience level of the operator.

We considered mild aortic valve prolapsed and mild aortic regurgitation is not an absolute contraindication to close the VSD with this device. This device has a soft and flexible design with a nitinol wire mesh layer. This device bends to the plane of aortic valve without interfering the coaptation of the aortic valves. Taking into account the risk of surgery and the limited cardiac surgery services in Indonesia, this less invasive technique can be used safely with proper pre intervention profiling of VSD by TTE and intraoperative LV angiography.

### Complete AVB and Other Complications

Complete AVB following transcatheter pmVSD occurred early during procedure or very late during follow-up leading to limited widespread use of this approach in many countries [5,6,7,20]. The incidence of AVB was lower using the duct occluder [13] and occluder from China [8,9,10,11] compared to the Amplatzer membranous occluder [5,6,7]. In the series, using ADO II [16,17] and Le VSD coil [12,13], no AVB was reported. Complete AVB did not develop in our study. Probably the Konar-MFO has less clamping force and radial stress property and has more flexibility and softer compared to the membranous VSD occluder, despite being less flexible and softer compare to ADO II and Le VSD Coil. These properties may reduce the trauma, compression or inflammation to the ventricular septum and conduction system. A recent study from Turkey using the Konar-MFO to close muscular and pmVSD reported complete AVB in 1% of their subjects [22].

Hemolysis, endocarditis or other complications did not develop during follow-up in this study. No mortality was noted until the latest follow-up. Furthermore, transcatheter pmVSD closure with the Konar-MFO provided additional advantages of abolishing the necessity of post-procedural intensive care and shortening hospital stay. Transcatheter pmVSD closure has a lower incidence of myocardial injury, less blood transfused, faster recovery, shorter hospital stay, and lower medical expenses [23]. Our patients did not require intensive care and most of them were discharged the day after procedure. These results are very important and promising in the setting of limited ICU beds such as in Indonesia.

To the best of our knowledge, this is among the first large multicenter study to evaluate the effectiveness and safety of the Konar-MFO to close pmVSD. This device also offered the variety of closure option approaches, simplicity of the procedure and probability to closure large defects in selected infants with congestive heart failure.

Practical consideration in preventing complications such as migration of the device and making implantation as safe as possible is proper patient selection including:

1. PmVSD with large inlet portion of the aneurysm (LV opening of the aneurysm) can be closed retrogradely as this approach may reduce much time.
2. If PmVSD and DCSA are immediately beneath the aortic valves (deficient aortic rims), we prefer to use the antegrade approach.
3. Malalignment VSD without aneurysm should not be closed with this device because of the risk of aortic sigmoid injury due to the symmetrical discs and the higher risk of device migration. However, this device can be considered to close the malalignment VSD with aneurysm and it has to be placed inside the aneurysm to avoid injuring the aortic valve via antegrade approach.
4. This device can be used for all type of pmVSD except for VSD with inlet extension of the aneurysm adjacent to tricuspid valves. The right disc of this device will protrude to the tricuspid valves and cause severe tricuspid regurgitation.
5. This device can also be used to close the DCSA VSD without malalignment of interventricular septum.
6. Closing the VSD with mild aortic prolapsed and mild aortic regurgitation can be challenging. However, this device can be considered to close this defect due to the soft and flexible design of this device. This device bends to the plane of aortic valve without interfering the coaptation of the aortic valves.
7. An endovascular snare kit can be used to evacuate the migrated device. The retrieval of MFO-KONAR were easier than other devices because of the soft design and no fabric patch attached on the nitinol wire mesh. Besides that, this device has a screw and a hub at the left and right disc. However, we had to change the delivery sheath to a large one (upsize 2 mm than the original one) in order to retrieve the device.

## Limitations

This observational prospective study did not include all patients of pmVSD closed with MFO. Our patients were also heterogeneous with wide range of age and size of the defect. However, the relatively large number of subjects included in this study provide encouraging evidence of the effectiveness and safety of transcatheter pmVSD closure using the Konar-MFO. Our findings of the effectiveness and safety of this device, which was based on a single-group observation, may need to be confirmed by another study with a longer follow-up because this study was limited to only one year follow up.

## Conclusions

Transcatheter closure of perimembranous VSD using the KONAR-Multi Functional Occluder is feasible, safe and effective, and has potential to use in selected infants with heart failure.

## Data Accessibility Statement

The data that support the findings are available from the corresponding author upon reasonable request.

## Figures and Tables

**Table:** 

ADO II	: Amplatzer Duct Occluder II
AVB	: atrioventricular heart block
AR	: aortic regurgitation
D	: the diameter of the left disc
D1	: the diameter of right disc
D2	: the diameter of base of the cone
JR	: Judgkin Right
LV	: left ventricle
MFO	: the KONAR-multi functional occluder
pmVSD	: perimembranous ventricular septal defect
PTFE	: polytetrafluoroethylene
RV	: right ventricle
TR	: tricuspid regurgitation
TTE	: transthoracic echocardiography
VSD	: ventricle septal defect

## Author Information

On behalf of the Cardiology Working Group, Indonesian Pediatric Society.
